# Electron Microscopy Structural Insights into CPAP Oligomeric Behavior: A Plausible Assembly Process of a Supramolecular Scaffold of the Centrosome

**DOI:** 10.3389/fmolb.2017.00017

**Published:** 2017-03-27

**Authors:** Ana L. Alvarez-Cabrera, Sandra Delgado, David Gil-Carton, Gulnahar B. Mortuza, Guillermo Montoya, Carlos O. S. Sorzano, Tang K. Tang, Jose M. Carazo

**Affiliations:** ^1^Biocomputing Unit, Macromolecular Structures, Centro Nacional de Biotecnología—CSICMadrid, Spain; ^2^Molecular and Computational Biology Program, Department of Biological Sciences, University of Southern CaliforniaLos Angeles, CA, USA; ^3^Structural Biology Unit, CIC bioGUNEDerio, Spain; ^4^Protein Structure and Function Program, Faculty of Health and Medical Sciences, Novo Nordisk Foundation Center for Protein Research, University of CopenhagenCopenhagen, Denmark; ^5^Institute of Biomedical Sciences, Academia SinicaTaipei, Taiwan

**Keywords:** centrosome, pericentiolar material (PCM), centriole, cilia, electron microscopy, negative staining

## Abstract

Centrosomal P4.1-associated protein (CPAP) is a cell cycle regulated protein fundamental for centrosome assembly and centriole elongation. In humans, the region between residues 897–1338 of CPAP mediates interactions with other proteins and includes a homodimerization domain. CPAP mutations cause primary autosomal recessive microcephaly and Seckel syndrome. Despite of the biological/clinical relevance of CPAP, its mechanistic behavior remains unclear and its C-terminus (the G-box/TCP domain) is the only part whose structure has been solved. This situation is perhaps due in part to the challenges that represent obtaining the protein in a soluble, homogeneous state for structural studies. Our work constitutes a systematic structural analysis on multiple oligomers of *HsCPAP*^897^^−1338^, using single-particle electron microscopy (EM) of negatively stained (NS) samples. Based on image classification into clearly different regular 3D maps (putatively corresponding to dimers and tetramers) and direct observation of individual images representing other complexes of *Hs*CPAP^897−1338^ (i.e., putative flexible monomers and higher-order multimers), we report a dynamic oligomeric behavior of this protein, where different homo-oligomers coexist in variable proportions. We propose that dimerization of the putative homodimer forms a putative tetramer which could be the structural unit for the scaffold that either tethers the pericentriolar material to centrioles or promotes procentriole elongation. A coarse fitting of atomic models into the NS 3D maps at resolutions around 20 Å is performed only to complement our experimental data, allowing us to hypothesize on the oligomeric composition of the different complexes. In this way, the current EM work represents an initial step toward the structural characterization of different oligomers of CPAP, suggesting further insights to understand how this protein works, contributing to the elucidation of control mechanisms for centriole biogenesis.

## Introduction

Centrosomes are found in most animal cells as the primary microtubule organizing centers (MTOC), being involved in important cellular processes, such as division, motility, and structural stabilization. This organelle is composed by two centrioles surrounded by a proteinaceous matrix of pericentriolar material (PCM), and is the base point for assembly o f cilia and flagella. Centrosomes serve as anchorage sites for a large number of regulatory processes (Doxsey et al., 2005) including the integration and activation of proteins that trigger cell division.

CPAP (also known as Centromere Protein J -CENPJ-, or as Sas-4 in *D. melanogaster* and SAS-4 in *C. elegans*) is a conserved and cell-cycle regulated protein (Tang et al., 2009) crucial for centrosome biogenesis. This versatile macromolecule plays roles in centriole elongation (by scaffolding cytoplasmic PCM complexes which posteriorly tether to centrioles; Zheng et al., 2014) and cilium disassembly (by providing a scaffold for the cilium disassembly complex, CDC; Gabriel et al., 2016); it also contributes to maintain spindle pole integrity (Chou et al., 2016). CPAP can be localized in different structures within the centrosome (Hung et al., 2000; Kleylein-Sohn et al., 2007) and in the cytoplasm (Schmidt et al., 2009).

It has been suggested that the N-terminal domain of CPAP acts as a scaffold for other cytoplasmic proteins, while its C-terminal domain tethers the new ensemble PCM complexes to the centriole, thus allowing to continue the formation of normal and functional centrosomes (Gopalakrishnan et al., 2011). Interestingly, the role of CPAP in the PCM assembly (through its N-terminus) seems to be independent of the role that it carries out along the formation of the centriole (through its C-terminus; Gopalakrishnan et al., 2011). It is hypothesized that the C-terminus of CPAP/SAS-4 localizes toward the interior of the centriolar A-tubule whereas the N-terminal part interacts with Cep152/Asterless and the neighbor microtubules (MTs; Cottee et al., 2013; Hatzopoulos et al., 2013). The multifunctional nature of CPAP and its interactions with different partners potentially implies a high level of structural complexity of this protein. At present, the oligomeric and structural dynamics of CPAP are poorly understood.

The C-terminus of CPAP includes a region known as the G-box or TCP domain, which is highly conserved through evolution, pointing to its primordial role in centriole/centrosome development. The determined crystallographic structures of the G-box/TCP domain revealed a solvent-exposed beta-sheet structure that remains stable by its own, despite lacking a hydrophobic core (Cottee et al., 2013; Hatzopoulos et al., 2013; Zheng et al., 2014). The rest of CPAP is formed by highly flexible regions and coiled-coil (CC) motifs (Hung et al., 2000), and its structure is not known; in human, the protein includes five CC domains. CPAP also contains both a MT binding domain (MBD, 423-607aa), which binds to a polymerized microtubule, and a tubulin-dimer binding domain (MDD, 311–422 aa) that binds to an αβ-tubulin heterodimer (Hsu et al., 2008) (Supplementary Figure 1).

In human CPAP (*Hs*CPAP), the region between residues 897–1338 (*Hs*CPAP^897−1338^) encompasses the G-box (residues 1150–1338), the coiled-coils CC4 and CC5 which together cover the residues 897–1056 (hereafter referred to as “CC4/CC5”), and an unstructured zone (residues 1066–1149, hereafter referred to as “CCGb-linker” region) making a linker between CC4/CC5 and G-box (Supplementary Figure 1A*). Mutations in the G-box and deletions within the CCGb-linker have been described in patients diagnosed with primary autosomal recessive microcephaly (MCPH) (Leal et al., 2003) or Seckel syndrome (SCKS) (Al-Dosari et al., 2010), respectively. Both syndromes present notable reduction in normal brain volume and intellectual disability, whereas maintaining the general architecture of a normal brain.

*Hs*CPAP^897−1338^ includes a number of distinct sites involved in the interaction of CPAP with other centrosomal proteins, such as P4.1R-135 (Hung et al., 2000), CEP135 (Vulprecht et al., 2012; Lin Y. C. et al., 2013), CEP120 (Comartin et al., 2013; Lin Y.-N. et al., 2013), STIL (Tang et al., 2011; Cottee et al., 2013), and 14-3-3 (Chen et al., 2006; Supplementary Figure 2). The CC5 domain of CPAP is sufficient to mediate its interaction with CDC (Gabriel et al., 2016) and is also required for the dimerization of CPAP (Zhao et al., 2010), the latter being important for maintaining centrosome cohesion. Indeed, the formation of CPAP oligomers as well as their disruption once the protein is phosphorylated during mitosis, is essential for the correct centrosome integration along the cell cycle (Zhao et al., 2010) and for regulation of interactions between CPAP and other proteins (Chen et al., 2006; Chou et al., 2016). Despite of the biochemical and biophysical evidences of CPAP dimer formation (Zhao et al., 2010; Hatzopoulos et al., 2013), there are neither clear structural data of such complex or other higher order oligomeric arrangements of CPAP. The current EM study of *Hs*CPAP^897−1338^ proves that this protein acquires several coexisting oligomeric states, showing striking new structural features that reveal the remarkably conformational versatility of this protein, which significantly contributes to the understanding of the biological role of CPAP.

## Materials and methods

### Plasmid construct

The full-length human cDNA of CPAP (1338 residues) was kindly provided by Professor Tang T. K., from the Institute of Biomedical Sciences, Academia Sinica. Taipei 115, Taiwan (Hung et al., 2000). The C-terminal residues 897–1338 of CPAP were amplified by PCR using a sense primer that incorporates the BamHI restriction site (5′-GCGGATCCCCTGGTGACAATGCTCG -3′), and an antisense primer containing BsrGI restriction site (5′-CGGTGTACATTACAGCTCCGTGTCCATTG -3′). To create a N-terminal 6xHis-tagged *Hs*CPAP^897−1338^ recombinant DNA construct (His-*Hs*CPAP^897−1338^), the amplified fragment was cloned into the BamHI-BsrGI site of the pST66Trc2-His expression vector (this is a pET3a modified plasmid created and kindly provided by Professor Song Tan, form the Center for Gene Regulation, Pennsylvania State University, State College, PA. EE. UU.), which allowed the incorporation of a six histidine tag (His-tag) to the N-terminus of the *Hs*CPAP^897−1338^ protein; in this way the recombinant expression plasmid pST66Tcr2-HisCPAP^897−1338^ was obtained. The strain *E. coli* DH5α was used as host for the cloning work. Purification of the recombinant plasmid was performed using standard methods (Green and Sambrook, 2012).

### Protein expression

*E. coli* BL21(DE3)pLysS cells were transformed with the pST66Tcr2-HisCPAP^897−1338^ recombinant plasmid, were grown at 23°C until an OD_600_ 0.6–0.8. Protein expression was induced by adding 0.2 mM IPTG to the cell culture, followed by incubation at 23°C/16 h. The cell pellet was resuspended in cold lysis buffer pH 7.4 (30 mM Na_2_HPO_4_, 20 mM NaH_2_PO_4_, 300 mM NaCl, 10% v/v Glycerol, 0.1% Triton X-100, 25 mM Imidazole, 10 μg/ml DNase A, 10 μg/ml RNase I, 0.5 mg/ml Lysozyme, 1X Protease Inhibition Cocktail EDTA-free. Sigma) at a temperature of 4°C, and lysed with a French Press. Solubility tests showed that *Hs*CPAP^897−1338^ protein is highly insoluble in low salt buffers, so it was purified and was maintained most of the time in the presence of 300 mM NaCl. Only part of the protein sample was subjected to dialysis in the presence of 150 mM NaCl prior to purification with ion exchange chromatography.

### Protein purification by immobilized metal affinity chromatography (IMAC)

After centrifugation (40,000 rpm/45 min) of the cell lysate, the soluble His-tagged protein in the clarified supernatant was purified by immobilized metal affinity chromatography (IMAC) with a Talon resin (Talon superflow. GE Healthcare) following the recommendations of the manufacturer. The collected fractions were analyzed by SDS-PAGE (Any KD Mini-PROTEAN TGX Gel. BIO-RAD), followed by staining with SimplyBlue SafeStain (Invitrogene); the CPAP identity of the observed protein bands was confirmed by Western Blot (against the His-tag) and Mass Spectrometry (MALDI TOF/TOF MS) analysis (data not shown).

### Size exclusion chromatography (SEC) analysis

Part of the protein samples eluted from IMAC (using a linear imidazole gradient) were concentrated and then loaded into either preparative or analytical Superdex 200 columns equilibrated with cold buffer H-300 (20 mM Hepes pH 7.4, 300 mM NaCl, 0.25 mM TCEP and 10% Glycerol) at a temperature of 4°C. The collected fractions were analyzed by SDS-PAGE (Any KD Mini-PROTEAN TGX Gel. BIO-RAD) stained with SimplyBlue SafeStain (Invitrogene). Some selected fractions from SEC were analyzed by NS-EM. Native-PAGE was used to determine the oligomeric state of flexible, non-globular particles.

### Ion exchange chromatography

The rest of the protein samples eluted from IMAC were pooled and dialyzed for 3 h at 4°C against buffer H-150 (20 mM Hepes pH 7.4, 150 mM NaCl, 0.25 mM TCEP and 10% Glycerol). During the dialysis process, *Hs*CPAP^897−1338^ formed thin fiber-like structures that could be detected at plain sight. NS-EM visualization of the sample before and after dialysis showed a heterogeneous set of particles in both cases; interestingly, only in the last condition, modular rope-like structures of variable length could be seen. In an attempt to obtain fractions enriched with the elongated supramolecular complexes, the dialyzed sample was then applied on an anion exchange HiTrap Q HP column (GE Healthcare Life Sciences) equilibrated in the same buffer. The collected fractions were analyzed by NS-EM.

### *In silico* structural analysis: sequence alignment and atomic structural modeling

Predictions of order/globularity and “disorder” (highly flexible; Janin and Sternberg, 2013) regions of *Hs*CPAP (full-length) were made using the GlobProt2 server (Linding, 2003). For *Hs*CPAP^897−1338^, the secondary structure was predicted with Jpred4 (Drozdetskiy et al., 2015); for the prediction of CC regions the web server COILS (Lupas et al., 1991) was used and, finally, the web servers PrOCoils (Mahrenholz et al., 2011) and LOGICOIL (Vincent et al., 2013) were employed to predict the oligomeric state probability of the CC sequence. The atomic modeling of CC4/CC5, CCGb-linker, and G-box regions of *Hs*CPAP, was carried out with I-TASSER (Iterative Threading ASSEmbly Refinement) (Zhang, 2008; Roy et al., 2010). The crystallographic structure of the G-box/TCP domain (PDB-4BXR), together with the structures modeled for the CC4/CC5 and CCGb-linker regions, were used for both, proposing a model of the *Hs*CPAP^897−1338^ monomer and making a coarse tentative fitting in the EM reconstructed maps of *Hs*CPAP^897−1338^ oligomeric complexes.

### Transmission electron microscopy

For negative staining (NS) of the samples, a drop of the protein solution was applied directly onto a glow-discharged EM grid (QUANTIFOIL. Formvar/Carbon. Cu 400 mesh grids), and allowed to be adsorbed on the grid surface (for few seconds or minutes, depending on protein concentration); then the drop was blotted with filter paper (Whatman grade No. 1) and the grid was washed by touching the surface with two consecutive drops of 0.75% (w/v) uranyl formate, blotting each time, and stained for 1 min with one more drop of the same staining agent. Finally, the grid was blotted again and allowed to air dry before observation.

### EM image acquisition, processing, analysis, and 2D image classification

NS-EM grids were examined in a JEOL JEM-1230 (accelerating voltage 100 kV) electron microscope, and images were recorded with a CCD camera ORIUS SC100 (4 × 2.7 k pixel) at 40,000x magnification. CTF corrected images were downsampled by a factor of 2 so the resulting image pixel size was 4.56 Å/pixel. All image preprocessing, particle selection and two-dimensional (2D) analysis steps were carried out following the general workflow of the image processing package Xmipp 3.1 (De la Rosa-Trevín et al., 2013). EM single particles were either manually or semi automatically selected (Abrishami et al., 2013). 2D class averages of particles were obtained using the clustering and alignment procedure CL2D (Sorzano et al., 2010).

### 3D map reconstruction and fitting of atomic structures

EM images of the *Hs*CPAP^897−1338^ samples were used to obtain several initial volumes, which were generated with Xmipp 3.1 using the Random Conical Tilt (RCT) method (Radermacher, 1988; Sorzano et al., 2015a) and the RANSAC algorithm (Vargas et al., 2014). The refinement of the volumes was performed by combining the use of the algorithms Projection Matching (Penczek et al., 1994) and Reconstruct Significant (Sorzano et al., 2015b) and the computer program Relion (Scheres, 2015), all of them accessed in the Scipion framework (De la Rosa-Trevín et al., 2016). The resolution of the 3D maps was determined using the “gold standard” Fourier Shell Correlation (FSC) 0.143 cutoff criterion (Van Heel and Schatz, 2005). The threshold level isosurface at which each density map was displayed to approximately represent the theoretical molecular mass was calculated with the program EMAN1 (Ludtke et al., 1999), which assumes a protein density of 1.35 g/ml (0.81 Da/Å^3^). Volume segmentation, coarse fitting of the atomic structures and the figures of the maps, were all done with UCSF Chimera (Pettersen et al., 2004).

## Results

### Sequence analysis and structural modeling

*In silico* analysis of *Hs*CPAP^897−1338^ secondary structure (Supplementary Figures 1A*, 3B,C) predicts three different regions distributed as follows: 38% is mostly formed by α-helices and corresponds to the CC4/CC5 region, 19% constitutes the unstructured CCGb-linker, and 43% shows several short beta-strands belonging to the G-box, which is in agreement with the solved structures of the equivalent domain of CPAP in *D. rerio* and *D. melanogaster* (Cottee et al., 2013; Hatzopoulos et al., 2013; Zheng et al., 2014). The secondary structure prediction obtained with Jpred4 was cross-checked using the programs PSSpred (Yan et al., 2013), SOPMA (Geourjon and Deleage, 1995), and APSSP2 (Raghava, 2002); comparable results were obtained in all the cases (data not shown).

**Figure 1:**
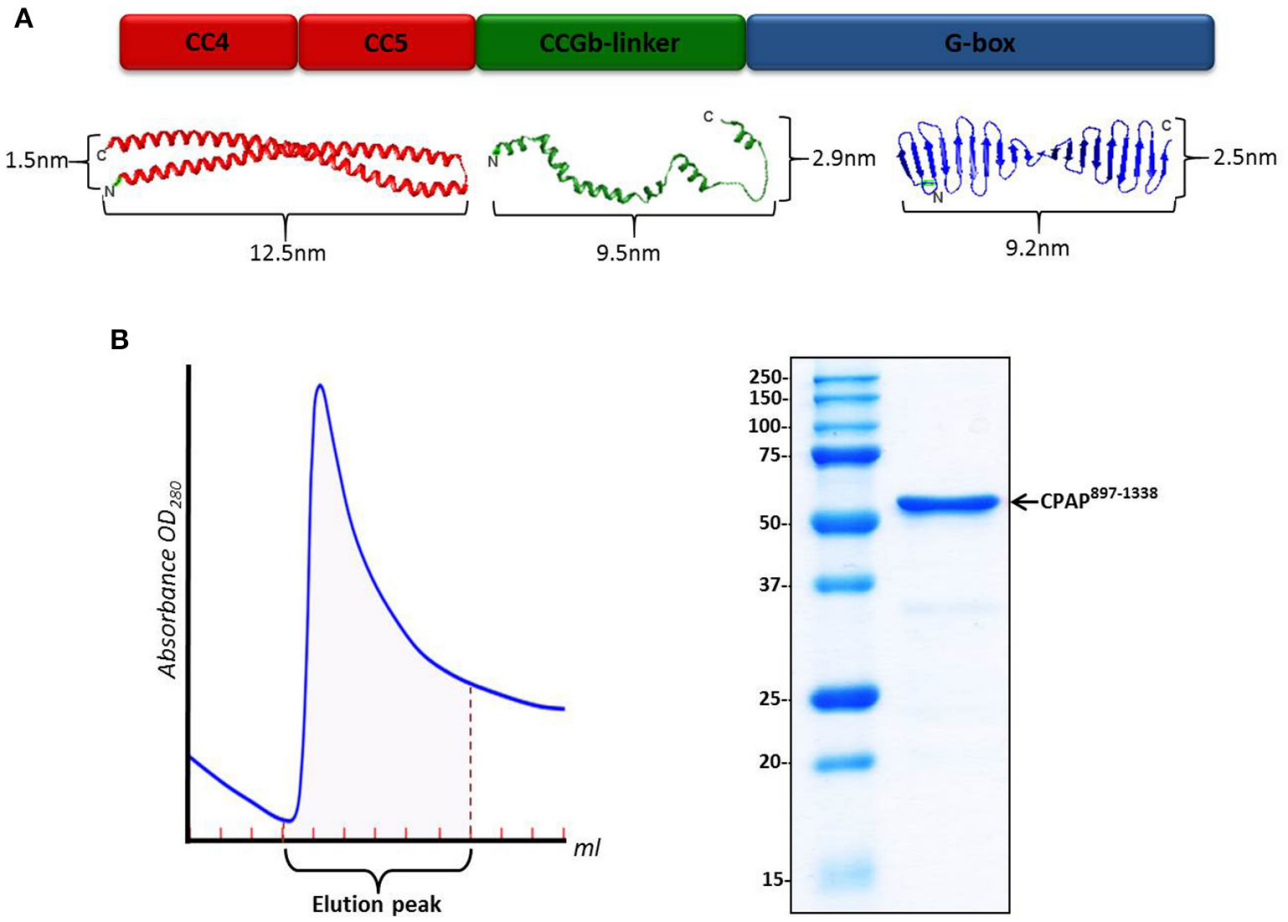
*****Hs***CPAP^**897−1338**^ construct. (A)** Color-coded schematic representation of the structural domains of *Hs*CPAP^897−1338^ (up) and their related atomic model (down). From left to right: I-TASSER predicted atomic models for the CC4/CC5 region (in red), an extended state version of the flexible CCGb-linker (in green), and the crystallographic structure of the G-box/TCP domain (PDB-4BXR) (in blue). The CCGb-linker region is expected to be very flexible, so its dimensions could vary significantly from the ones shown here. **(B)** Chromatographic elution profile (left) of the immobilized metal affinity chromatography (IMAC) purification of *Hs*CPAP^897−1338^ and SDS–PAGE (right) of the pooled fractions from the elution peak.

Atomic models for the different domains of *Hs*CPAP^897−1338^ were obtained using I-TASSER (Figure 1A and Supplementary Figure 3D), which generates 3D models by multiple-threading alignments and iterative structural assembly simulations, using templates from the Protein Data Bank (PDB) that it identifies as potential structural homologs (Supplementary Table 1). The structural modeling of CC4/CC5 shows a single CC formed by two antiparallel α-helices, each of ~12.5 nm, where CC4 corresponds to the N-terminal α-helix and CC5 to the C-terminal one. When modeling CC4 and CC5 separately, the structure of an extended α-helix was obtained on each case (data not shown); this agrees with the model of a long CC formed by CC4/CC5, instead of two consecutive, short CCs. Considering that the predicted CC4 and CC5 are virtually one after the other, without a clear long separation in between, it is possible that CC4/CC5 behaves as a single CC. Although the CCGb-linker region is predicted to be intrinsically flexible (meaning that there is not a unique/fixed structure), we still include the analysis of some possible structures modeled by I-TASSER, as a guide of the general dimensions for potentially contracted or extended states of this zone of the protein. Since the model obtained for the G-box looked almost the same as the atomic structure of the PDB-4BXR (G-box/TCP domain from *D. rerio*; Supplementary Figure 3D), whose sequence has ~68% identity with the G-box from human, we decided to use the crystallographic structure in the further steps of our analysis. In human and *D. rerio* the part of CPAP comprising CC4/CC5, CCGb-linker and G-box domains is well conserved with 50% sequence identity (Supplementary Figure 3A).

### Protein expression and purification and identification of diverse homo-oligomeric forms

In our hands, the full-length *Hs*CPAP protein proved to be highly insoluble (data not shown). Similar to many other centrosomal proteins (Dos Santos et al., 2013; Treviño et al., 2014), CPAP is predicted to be mostly comprised of flexible and CC regions (Hung et al., 2000; Supplementary Figure 1), which are two hallmark characteristics commonly associated with insoluble samples, difficult to handle and purify.

The sequence of *Hs*CPAP^897−1338^ codes for a protein with a theoretical molecular weight (MW) around 52 kDa. This construct includes the only globular domain of CPAP (the G-box); the remaining 57% of its sequence is composed by the CC4/CC5 region and the predicted non-structured region CCGb-linker (which connects CC4/CC5 with the G-box). Our N-terminal 6xHis-tagged *Hs*CPAP^897−1338^ construct is partially soluble after optimized expression and purification conditions, which included using a buffer salt concentration of 300 mM NaCl to avoid protein aggregation (see details in Section Materials and Methods). A single step IMAC purification yields a pure protein sample (Figure 1B) which, after concentration, was subjected to size exclusion chromatography (SEC).

We faced problems obtaining reliable measurements of the protein concentration using both colorimetric (Bradford assay) and spectrophotometric (absorbance at 280 nm) methods. In the first case, very low and inconsistent measurements were obtained, possibly because of inefficient binding of the dye to the protein due to the low number of aromatic residues in *Hs*CPAP^897−1338^ (i.e., there are only two tryptophans), resulting in an underestimation of the protein concentration. On the other hand, the IMAC purified samples eluted with a linear imidazole gradient were directly passed through SEC columns (without previous buffer exchange to eliminate the imidazole since we observed evidence of protein precipitation during the dialysis process), so the precise concentration of imidazole was difficult to calculate. Since imidazole absorbs UV radiation at 280 nm (in proteins, the absorbance at 280 nm is primarily due to the presence of the amino acid tryptophan followed by tyrosine) it was not possible to make a reliable spectrophotometric determination of the protein concentration.

Variable SEC profiles obtained in a number of purifications of *Hs*CPAP^897−1338^ performed under the same buffer and temperature conditions (Supplementary Figure 4), indicated differences in size and abundance of the macromolecular specimens present. A possible explanation to this phenomenon is the change in concentration of the initial input samples passed through each of the SEC runs (as visualized by comparing the density of the protein bands in SDS-PAGE), which could suggest the possibility that the concentration of the protein is a factor influencing the oligomerization state of *Hs*CPAP^897−1338^, something that has been reported to occur in other centrosomal proteins rich in CC motifs and non-structured regions (Treviño et al., 2014). The protein concentration-dependent oligomerization behavior of the G-box domain (Hatzopoulos et al., 2013; Cutts et al., 2015), supports our observations.

The relatively broad elution profile obtained in some cases (Supplementary Figure 4A) is explained by the coexistence of *Hs*CPAP^897−1338^ particles of different hydrodynamic radius (shape/size) in the sample; this was confirmed by EM analysis of the eluted fractions. A possible dynamic equilibrium between different-sized complexes could also contribute to form a relatively broad elution profile.

### Structural characterization of different *Hs*CPAP^897−1338^ homo-oligomeric complexes and coarse fitting of atomic models

Visualization of NS-EM images of a number of *Hs*CPAP^897−1338^ purified samples allowed us to identify and characterize the structure of different oligomeric complexes formed by this ~52 kDa protein construct. Three main forms of *Hs*CPAP^897−1338^, each corresponding to a different oligomeric state, eluted from a broad SEC (Superdex 200 10/300) peak (Supplementary Figure 4A) showing that distinct oligomeric states of the protein can coexist under the tested experimental conditions. Other SEC runs showed a more defined peak for some of the oligomeric forms (Supplementary Figures 4B,C). The first type of particle consisted of flexible, small rope-like structures found in a wide range of elution volumes, which seem to concentrate mostly in SEC fractions along a range approximately between 120 and 170 kDa. Due to the flexible shape of these fibrillary particles their hydrodynamic behavior may vary dynamically, differing significantly from their actual molecular weight and producing the observed broad eluting profile, indicative of multiple different extended, or more contracted conformations that these flexible strings adopt. It is likely because of this flexible behavior that it was not possible to completely isolate the fibrillary particles from the more globular ones by using SEC. A second class of particle, which elutes around an apparent MW of ~108 kDa, showed a toroid-like shape. The last type of particle presents a barrel-like shape and elutes at an apparent MW of ~208 kDa. The difficulties obtaining reliable measurements of the protein concentration hindered us to make a quantitative analysis to determine precise concentration-dependent changes in the morphologies of the oligomers.

A putative assignment of the oligomeric states was performed based on elution profiles, native gels, EM images and maps, and a very coarse fitting of a number of copies of the atomic model of *Hs*CPAP^897−1338^. It is important to highlight that these fittings are only intended to show how the different maps are compatible with the oligomeric state assigned to them, and not to depict precise quasi-atomic arrangements. Nevertheless, some general structural features provided by the EM data have allowed us to suggest a tentative global arrangement of the protein domains.

#### Flexible, fibrillary particles

**Figure 2:**
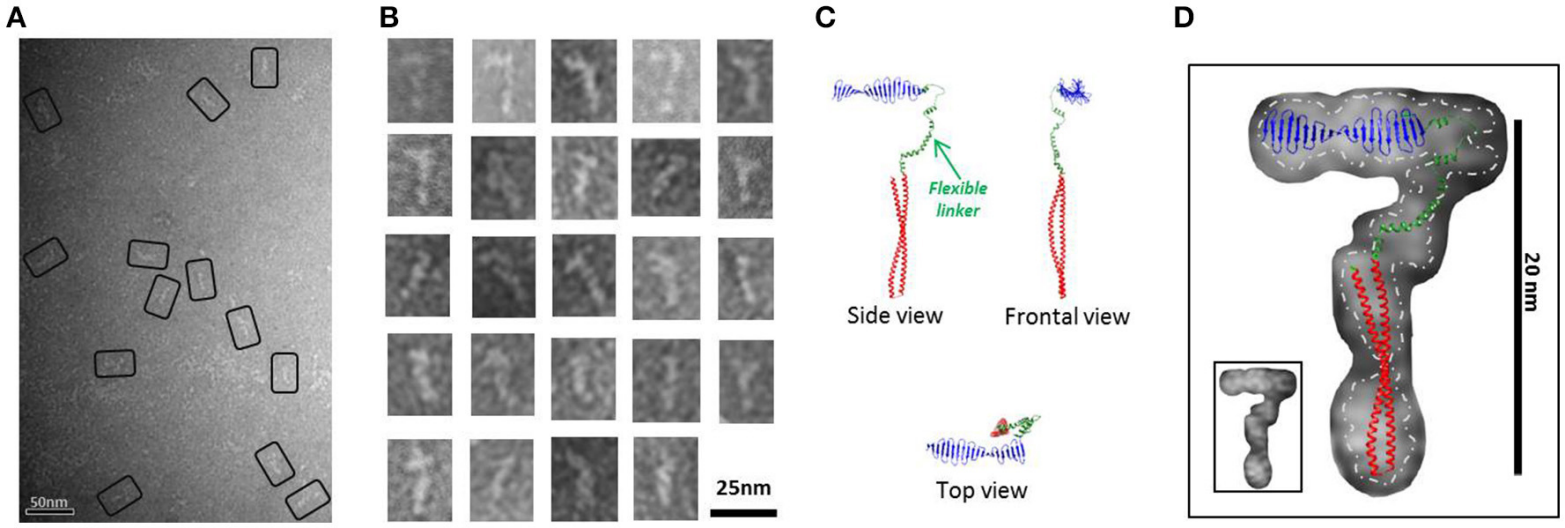
*****Hs***CPAP^**897−1338**^ flexible fiber-like particles. (A)** Micrograph of a SEC sample eluted between apparent 120-170 kDa (see Supplementary Figure 4B) showing a population of flexible fiber-like particles. Some selected particles are highlighted by black rectangles. **(B)** Windowed raw images of the particles show a general elongate cane-like structure. **(C)** Proposed *Hs*CPAP^897−1338^ monomer model, assembled using the predicted structural for CC4/CC5 and CCGb-linker domains (see Figure 1A) and the X-ray structure of the G-box (PDB-4BXR). **(D)** Superimposition of the monomer model (from **C**) on a representative single particle image of a *Hs*CPAP^897−1338^ fibrillary particle. Fitting of the atomic model with an inverted orientation of the domains, compared to the one showed in the figure, is also feasible. A discontinuous line demarks the contour of the EM particle, separating it from the background; a smaller version of the same particle is shown in the inset at the lower left corner.

A native PAGE of a SEC (Superdex 200 16/60) purified peak at elution volume of 64–70 ml (between apparent 120 and 170 kDa; Supplementary Figure 4B) enriched by *Hs*CPAP^897−1338^ small fibrillary particles (Figures 2A,B), was performed to determine the oligomeric state of these flexible strings, showing that the predominant state of this sample was monomeric (observed as a thick dense band close to the 66 kDa marker) and that only a negligible part formed higher order oligomers. Western blot against the N-terminal His-tag of the protein revealed that the small amount of protein running as lower molecular weight bands (below the 66 kDa marker) corresponded to degradation products occurring by the *Hs*CPAP^897−1338^ C-terminal side (data not shown). CPAP identity of all the observed bands was confirmed by mass spectrometry (MS).

A 2D structural analysis of some of these flexible particles was made by measuring the general dimensions of the most representative images, which presented a cane-like shape with a “shaft” of ~15–17 nm long and a flexible “handle” of ~10 nm long. To give additional support to the assignment of a putative monomeric state to these flexible particles, a complete atomic model for a possible *Hs*CPAP^897−1338^ monomer was assembled. We combined the crystallographic structure of the G-box/TCP domain (PDB-4BXR) and the models of the CC4/CC5 and the CCGb-linker (which is in a relatively extended conformation) regions, as predicted by I-TASSER (Zhang, 2008; Roy et al., 2010); here, the CCGb-linker acts as a flexible hinge that connects the G-box and CC4/CC5 domains (Figure 2C). We observed that the general dimensions and the shape of some of the fiber-like EM images fitted quite well with a monomer of the proposed model (Figure 2D).

#### Toroidal complex

**Figure 3:**
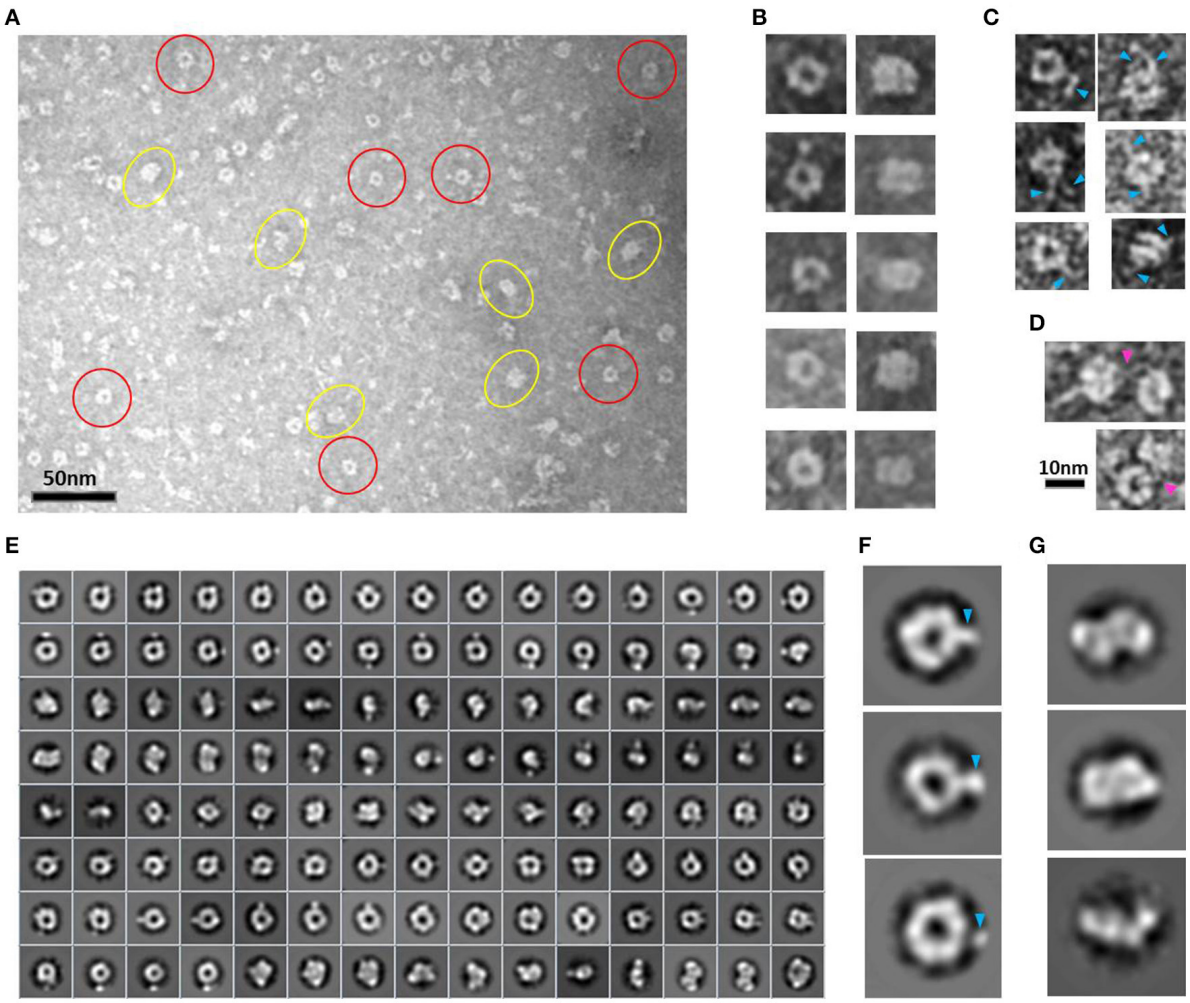
*****Hs***CPAP^**897−1338**^ toroidal particles. (A)** Micrograph of a SEC sample eluted at an apparent MW of ~108 kDa, compatible with a dimer (see Supplementary Figure 4A), showing mostly toroidal (highlighted by red circles) and a more rectangular kind of images (highlighted by yellow ovals). **(B)** Windowed raw images of representative particles proposed to be top views (left panel) and lateral views (right panel) of the same type of particle. **(C)** Single particles showing extended flexible projections (pointed by blue arrowheads). **(D)** Pair of particles interacting through flexile strings (pointed with a fuchsia arrowhead). **(E)** Reference-free class average image classification (CL2D). **(F)** Toroidal class images showing an extra mass at the periphery (pointed by a blue arrowhead). **(G)** 2D class images of more elongated and flat particles that could correspond to intermediate conformations of the final dimer.

EM analysis of several consecutive fractions eluted from a SEC (Superdex 200 10/300) purification of *Hs*CPAP^897−1338^ (Supplementary Figure 4A), revealed that a fraction eluted at an apparent MW of ~108 kDa, which is compatible with the size of a dimer, was mostly populated by both images of tori with an external diameter of ~10 nm and a more rectangular kind of particles with a size of ~7.5 nm (*height*) × ~10 nm (*width*), which look like two wavy threads bound by their wider sides (Figures 3A,B). Some of these particles showed one or two thin flexible strings protruding to the outside (Figure 3C) and, interestingly, we observed pairs of particles joint through these flexible extensions (Figure 3D), suggesting their role in the formation of a higher-order multimer, a putative tetramer.

During the averaging of the aligned images, the signal of highly flexible structures tends to be lost, although the signal of the base part uses to remain visible for being a most stable point. Indeed, in agreement with the aforementioned observations, the 2D class average classification of 36,699 single particle images (Figure 3E) showed some toroidal particles presenting a punctual, strong extra density at the periphery of the ring (Figure 3F), which could be attributed to the average of the base part of the flexible strings observed in the single particle images (Figure 3C), although it cannot be excluded that more compact conformations of the flexible projection exist. 2D image classification revealed the presence of other more elongated and flat particles (Figure 3G), some of which looked like two small consecutive rings or an infinity symbol (∞), suggesting a coexistence of putative dimeric intermediate conformations.

**Figure 4:**
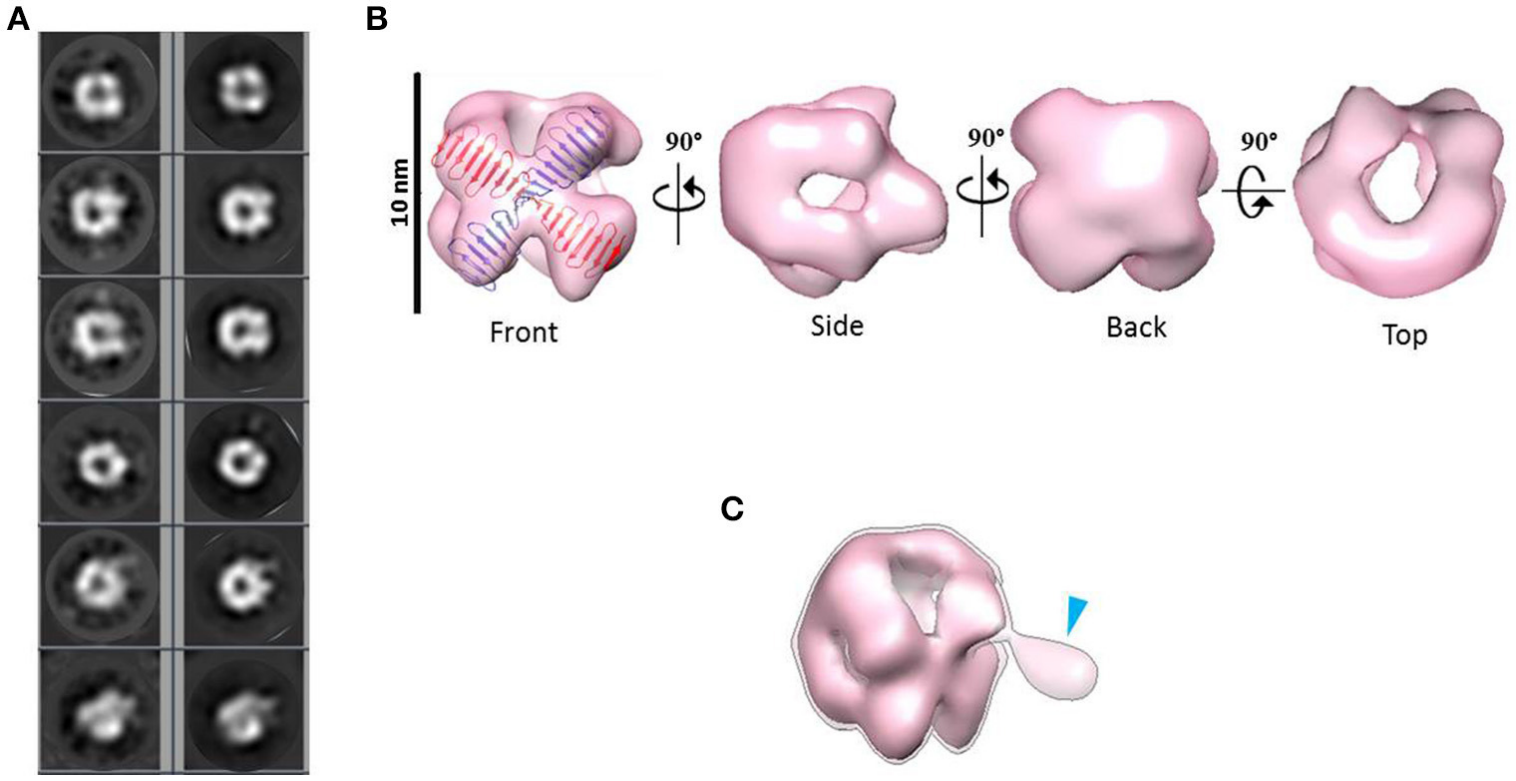
**3D reconstruction of a putative ***Hs***CPAP^**897−1338**^ homo-dimeric complex. (A)** Reference-free class averages (left column) and the corresponding forward projections (right column) of the 3D reconstruction. **(B)** Different views of the putative *Hs*CPAP^897−1338^ dimer with two copies of the atomic coordinates of G-box/TCP domain (PDB-4BXR, represented in blue and red) fitted in the cross brace-like frontal face. **(C)** Tilted view of the 3D map displayed at a lower contour level (shown as a semi-transparent density) than in **(B)** where it is observed an extra mass (pointed by a blue arrowhead).

Combining 2D and 3D classification image processing methods allowed us to obtain a final 20 Å resolution map (using the FSC = 0.143 criterion) of an asymmetrical toroid-like structure (Figure 4) containing 4,587 particles out from the initial data set. This structure represents the most frequent kind of particles (Figure 3B) of the putative dimer of *Hs*CPAP^897−1338^, although the 3D classification revealed some degree of global flexibility of the structure. What we called the frontal view of the complex (Figure 4B) shows a slightly twisted cross brace structure where the length of each of the two crossed lines is around ~9.2 nm, which matches the length of the G-box, however, it is too short to fit the model of CC4/CC5. Two copies of the crystallographic structure of the G-box/TCP domain (PDB-4BXR) can be reasonably fitted in the frontal face of the map (Figure 4B, first panel at the top left corner). Compared with the frontal view, the opposite side (back view) presents a less well defined X shape, more bulky and convex, making a rigid fitting more challenging. Two copies of an arched version of the CC4/CC5 model could be accommodated on the backside of the map in an X shape disposition if extending each domain toward one of the densities of each of the sides. Finally, each of the four remaining sides has an elongated, irregularly-shaped pore. Fitting of two copies of the CCGb-linker in the remaining density along the sides of the map seems possible if using a more contracted version of this flexible domain, compared with the model showed in Figure 1A.

When the 3D map is displayed at a lower contour level (Figure 4C), an additional density emerges from a corner of the frontal face of the structure, which may correspond to the basal part of one of the flexible strings observed in some of the single particle images (Figures 3C,F). The proposed tentative location of two copies of the G-box in the frontal face of the map, with the remaining part of each of the monomers around it, would suggest that this flexible protrusion would putatively correspond to the most N-terminal segment of *Hs*CPAP^897−1338^. Considering the aforementioned proposal, together with the interacting putative dimers showed in Figure 3D, one possibility is that while intermolecular associations via the CC5 domain of the monomers stabilize the dimer, the CC4 domain could extend temporarily acting as a brooch allowing an initial approach to a second dimer, with the subsequent formation of the tetramer.

#### Barrel-meshed like complex

**Figure 5:**
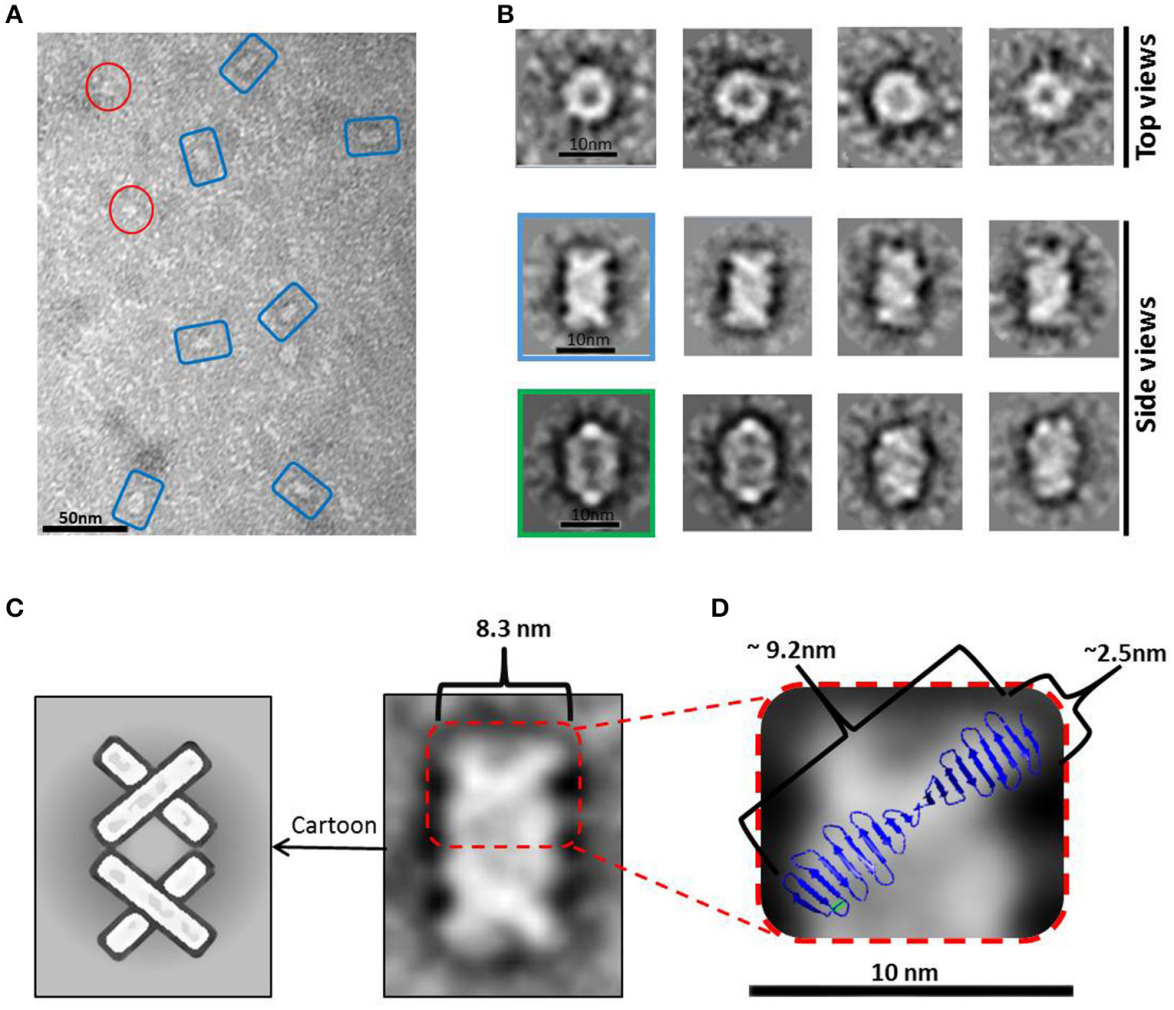
*****Hs***CPAP^**897−1338**^ barrel-like complex. (A)** Micrograph of a SEC sample eluted at an apparent MW of ~208 kDa, compatible with a tetramer (see Supplementary Figure 4C), showing what could correspond to side and top views of barrel-like particles (highlighted by blue rectangles or red circles, respectively). **(B)** Reference-free class averages of top views (panel of the first row) and side views (panels of the last two rows) of the putative *Hs*CPAP^897−1338^ tetramer complex. Some side view images are almost rectangular and narrower (image highlighted in blue) while other kind of images are wider and more oval-shape (image highlighted in green). **(C)** 2D class average image of a rectangular side view of 8.3 nm width (right), which resembles two stacked crossbars (see schematic representation on the left panel). **(D)** Zoom (x4) of the upper half of the 2D class image in **(C)**, where the superimposition of the atomic structure of *D. rerio* G-box/TCP domain (PDB-4BXR) shows the match between the dimensions of a diagonal string (~2.5 × ~ 9.2 nm) in the particle and the ones of the crystallographic structure of the G-box/TCP domain.

A peak fraction from a gel filtration (Superdex 200 10/300) purification step of *Hs*CPAP^897−1338^ eluted at an apparent MW of ~208 kDa (Supplementary Figure 4C), which is compatible with the size of a tetramer, was analyzed by NS-EM (Figure 5A). Reference-free average classification of 3,048 particles showed a meshed structure around 14.5 nm length, that presents two different types of side views, each around 9.3 and 8.3 nm maximum width (Figure 5B). The wider side view has an ellipsoid shape flanked by two marked, round electron densities and traversed by two bent bridges. The narrowest side view looks like a lattice formed by four strings with a diameter around 2.5 nm, which agrees with the diameter of the crystallographic structure of the G-box/TCP domain (PDB-4BXR; Figures 5C,D). We also noted that the ~9.2 nm length of the atomic structure of the G-box matched well with the longitude of each of the four diagonal lines forming pairs of cross brace arrangements in some of the 2D average classes (Figure 5D). Four copies of the G-box can be accommodated by aligning each molecule with one of the elongated densities observed in the 2D class showed in Figure 5C. Interestingly, each transversal half from the above mentioned 2D class of the putative tetramer (Figures 5C,D), resemble the shape and size of the cross brace-like frontal face in the 3D map of the putative dimer of *Hs*CPAP^897−1338^ (Figure 4B).

**Figure 6:**
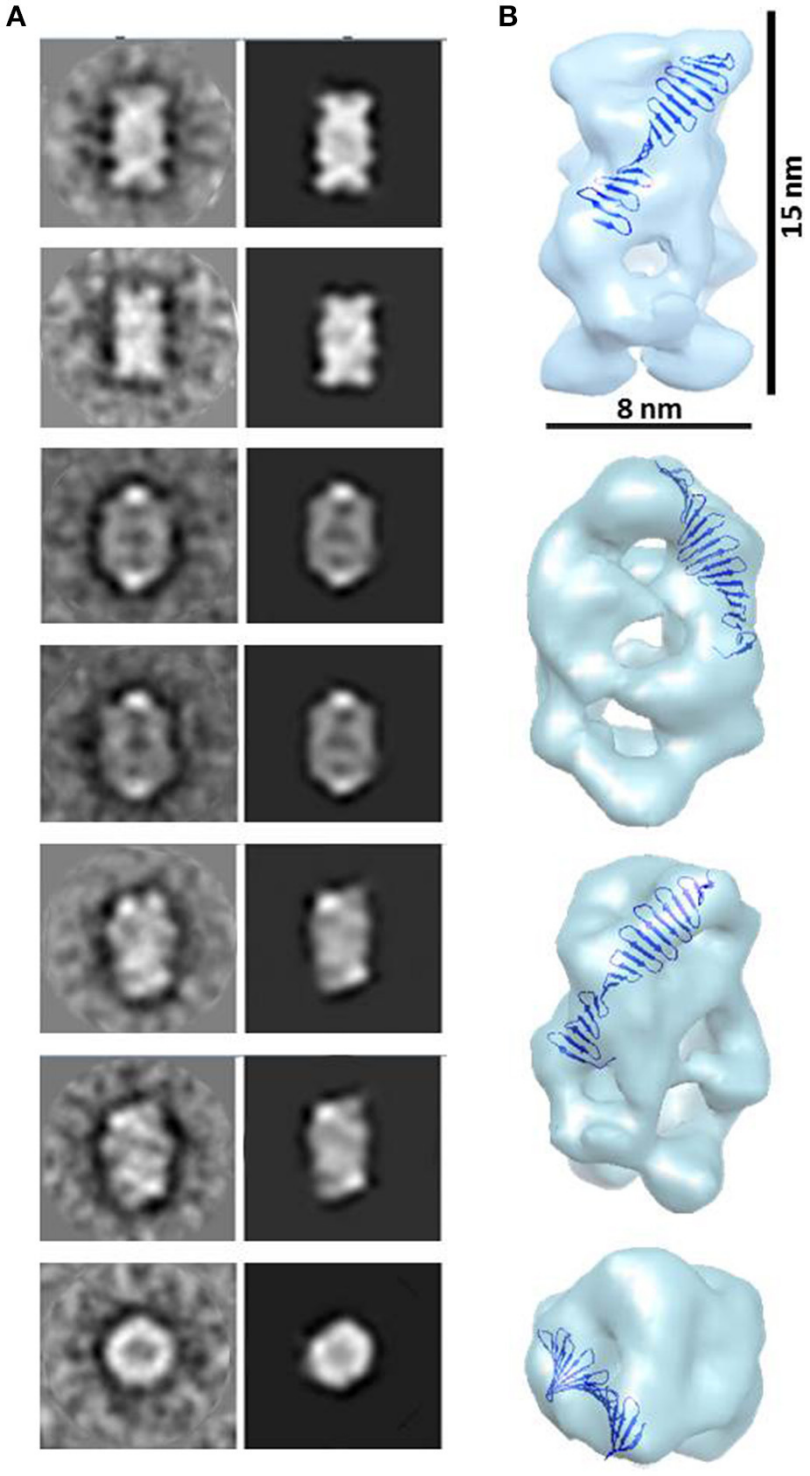
**3D reconstruction of a putative ***Hs***CPAP^**897−1338**^ homo-tretrameric complex. (A)** Reference-free class averages (left column) and the corresponding forward projections (right column) of the 3D reconstruction. **(B)** Different orientations of the putative *Hs*CPAP^897−1338^ tetramer volume, together with a tentative fitting of the atomic structure of the G-box/TCP domain showing how the general dimensions of this structure fit into the EM volume.

A 23 Å resolution 3D map (using the FSC = 0.143 criterion) of the putative *Hs*CPAP^897−1338^ tetramer reveals the structure of a hollow, asymmetrical barrel-like complex made of intertwined strings. In line with the 2D analysis showed in Figures 5C,D, a tentative fitting of the atomic coordinates of the G-box (PDB-4BXR) shows how the general dimensions of this domain fit in our 3D EM map (Figure 6).

Highly flexible regions, as the CCGb-linker of CPAP is predicted to be (Supplementary Figure 3B), are often found as ductile linkers, allowing the protein to acquire different conformations. An alternatively proposed conformation of *Hs*CPAP^897−1338^ (a different conformation to the one presented in Figure 2C) where the CC4/CC5 domain bends toward the G-box producing a triangular-shape structural arrangement (Supplementary Figure 5A), allowed a coherent fitting of four monomers inside the volume of our putative *Hs*CPAP^897−1338^ tetramer (Supplementary Figures 5B,C). A tentative gross fitting has been done by distributing the structural domains of each of the monomers into the four longer sides of the density map, in the following way: four copies of the crystallographic coordinates of the G-box were fitted into one of the narrow lateral views (henceforth called the G-face), whose structure resembles the cross brace organization observed in the 2D class showed in Figures 5C,D; four copies of the atomic model of CC4/CC5 were fitted on the opposite side (henceforth called the CC-face), which shows a bulky lattice formed by pronounced elongations allowing to accommodate the four units; finally, two copies of the CCGb-linker model were accommodated into the two central, twisted bridge-like densities at each of the two wider sides of the map (henceforth called Linker-face1 and linker-face2). The fitting described above supports the proposed tetrameric state of this complex. It is worth mentioning that the proposed distribution of the domains within the density map creates a clear structural polarity where the G-face localizes opposite to the CC-face. Perhaps this could be a strategy to allow the CPAP oligomer to interact with different partners, either simultaneously or at different times.

It is noteworthy that the 3D map of the putative *Hs*CPAP^897−1338^ dimer presents both dimensions and shape compatibility with half of the 3D map of the putative tetramer. The above mentioned consideration, together with EM images showing two putative dimers interacting with each other (Figure 3D), strongly suggest that the ~208 kDa barrel-like complex (putative tetramer) may be formed by dimerization of the ~108 kDa toroidal complex (putative dimer).

#### Modular higher order supramolecular rope-like structures

**Figure 7:**
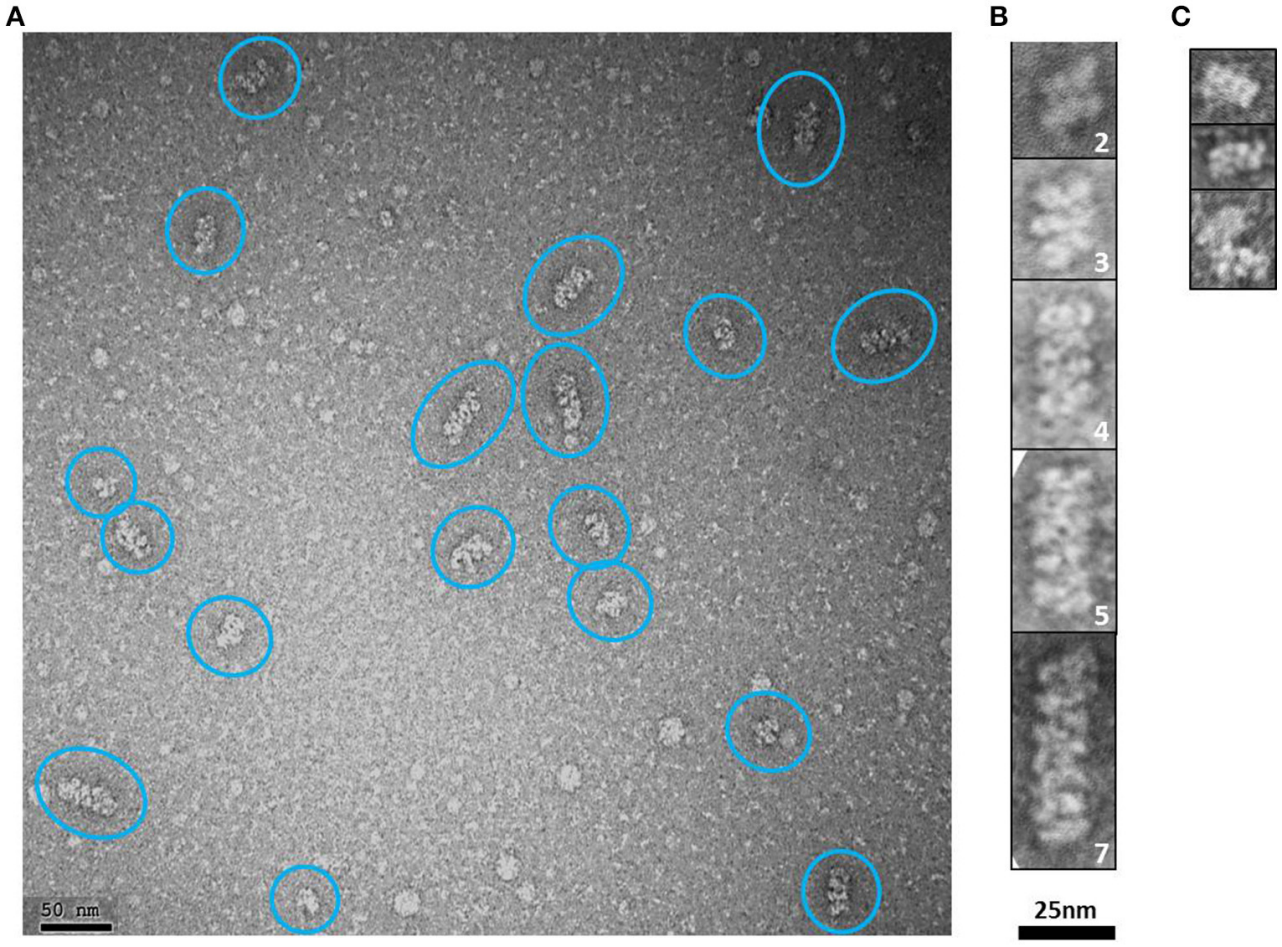
*****Hs***CPAP^**897−1338**^ putative tetramers may stack together by their longer sides to form modular higher order complexes of variable length. (A)** Representative negative stain electron micrograph of an anion exchange purified *Hs*CPAP^897−1338^ fraction, where there are observed higher order modular-like complexes of different length (highlighted by blue ovals). **(B)** Windowed zoomed images of some selected particles where the number of stacked modules is indicated by the number on the lower right corner of each image. **(C)** Images of individual modules (first two rows) or partially interacting modules (last row).

Elongated rope-like structures were formed during the dialysis process of lowering the buffer salt concentration from 300 mM NaCl (buffer H-300) to 150 mM NaCl (buffer H-150; see Section “Ion Exchange Chromatography” from Section Materials and Methods). The dialyzed sample was further subjected to an anion exchange purification step. NS-EM of protein fractions eluted between 350 and 450 mM NaCl from the anion exchange column, showed modular rope-like structures of different lengths. At first glance, these structures seem to be formed by linear stacks of a variable number of rectangular modules with dimensions varying between ~7–8.5 nm (*length*) × ~15–16 nm (*width*) (Figures 7A,B). Interestingly, the dimensions of completely or partially isolated blocks found in this sample (Figure 7C), match with those of the putative tetrameric complex. Therefore, it seems reasonable to suggest that the putative tetramers may self-assemble into organized, modular rope-like structures with an axial repeat of ~8 nm. A possible explanation for the slightly variable internal organization of the elongated, modular structures is that their building blocks have some intrinsic flexibility, causing small differences in length and width among modules. Another possibility is that the relative organization of the modules is not perfectly parallel (side by side) but instead presents a subtle disjointed disposition (e.g., helicoidal-like), which could produce the observed internal variations in the 2D projections of these modular structures.

It has been proposed that higher order assemblies of CPAP could act in a synergistic way as a platform to provide an interface mediating the tethering of PCM to the centriole (Gopalakrishnan et al., 2011; Hatzopoulos et al., 2013; Zheng et al., 2014). Our 3D-EM reconstruction of a putative *Hs*CPAP^897−1338^ tetramer and the observed elongated, modular structures with an ~8 nm axial periodicity, confirms that CPAP is able to form higher order assemblies.

## Discussion and conclusions

### *Hs*CPAP^897−1338^ forms different homo-holigomeric complexes *in vitro*

In this study we report that *Hs*CPAP^897−1338^ construct assembles into different coexisting homo-oligomeric complexes. Taking together the analysis of our biochemical and biophysical data, the 2D images and the 3D volume obtained by the EM work, the solved crystallographic structure of the G-box domain and, finally, the *in silico* structure modeling of the CC4/CC5 region and a gross estimation of the dimensions of the CCGb-linker of CPAP, we propose that this protein is highly flexible as a monomer, and that it presents a discrete but dynamic oligomeric behavior that includes the assembly of dimers, tetramers, and larger structures formed by stacks of tetramers. The predicted structural flexibility of the CCGb-linker could be an important factor in explaining how CPAP is able to acquire the different conformations that could be required to assemble into the observed oligomeric species.

Taking into account the multiple binding partners of CPAP, it is not surprising that a considerable part of this protein is predicted to be largely unstructured, being this a characteristic that can confer the structural flexibility necessary to interact with different proteins/complexes (Dunker et al., 2005). Indeed, it is reasonable to consider that each of the oligomeric states of CPAP would be more prone to establish an interaction with a specific protein/complex depending on its own initial configuration; the building up of different binding sites at the inter subunit interfaces of the different CPAP complexes could be a possible mechanism contributing to this.

It is well-known that concentration can be a driving factor affecting the oligomerization status of many proteins, which in turn, modifies its basic structure and, subsequently, its function (Giese and Vierling, 2002; Chen et al., 2003; Kutter et al., 2007; Kley et al., 2011). There exist reports of proteins forming filaments in response to factors such as the increment in concentration (Noree et al., 2010), in some cases, showing a modular behavior (Petrovska et al., 2014) resembling the one we observe in *Hs*CPAP^897−1338^. Indeed, it has been shown that the G-box domain forms large assemblies in a protein concentration-dependent manner (Hatzopoulos et al., 2013; Cutts et al., 2015). CPAP expression is regulated along the cell cycle and its levels increase gradually from the beginning of S phase until mitosis, this being a lapse of time that coincides with the procentriole formation (in early S phase), elongation (in late S phase), and with centrosome maturation (along the G2 phase). Remarkably, centrosomal CPAP maintains a continuous exchange with a cytoplasmic CPAP pool (Kitagawa et al., 2011), reaching its highest level in G2, when there is a maximal recruitment of proteins to the PCM. CPAP shows a significant decrease at the end of mitosis/early G1, when the dynamic of formation and maturation of the centrosome is already finished (Azimzadeh and Bornens, 2007; Tang et al., 2009; Kim et al., 2012). Furthermore, the CPAP protein level is also regulated during centriole amplification in multiciliated cells (Zhao et al., 2013). Protein concentration-dependent conformational changes could be a mechanism contributing to direct the network of diverse interactions that CPAP must carry out. Thus, it is tempting to speculate that cell cycle regulation of CPAP concentration could be a complex and highly synchronized strategy, which along with other determinant factors [e.g., protein phosphorylation (Chen et al., 2006; Chang et al., 2010; Zhao et al., 2010; Chou et al., 2016) or pH changes (Treviño et al., 2014)] controls the oligomeric state of CPAP in order to direct some of its multiple functions.

CPAP/SAS-4 has been localized in the proximity of the lumen centriole surface of microtubules (Kleylein-Sohn et al., 2007; Lawo et al., 2012; Mennella et al., 2012). It has been shown that CPAP interacts with the 8 nm length αβ-tubulin heterodimers (Hung et al., 2004) that form centriolar MTs. Column-like structures with a repeating unit around 8 nm have been observed at the luminal side in two recent tomographic reconstructions (Li et al., 2012; Guichard et al., 2013), and it has been suggested that, in part, they could be formed by CPAP. Furthermore, based on these data, Hatzopoulos et al. (2013) have proposed a model in which part of the C-terminal domain of CPAP (CTD) (more precisely, the G-box) is forming an elongate structure with ~8 nm periodicity in the luminal side of the triplets, close to the A-microtubule, while the CC region of the protein stretches toward the beginning of the C-microtubule. Here we show that *Hs*CPAP^897−1338^ forms column-like structures with an axial repetition pattern ~8 nm. Therefore, it can be suggested that this column-like structure, putatively formed by the tetramer of our CPAP construct, is the one making part of the 8 nm axial repeat structure in the centrosome lumen. Similarly to the model proposed by Hatzopoulos et al., it could be suggested that the remaining N-terminal part of the full length protein (residues 1–896 in *Hs*CPAP) extends away from the elongated modular structure formed by *Hs*CPAP^897−1338^.

It has been reported that the whole structure and protein composition of centrosomes is poorly affected by NaCl and KCl at concentrations as high as 2M (Klotz et al., 1990). Notably, we observed that the elongated modular structures of CPAP (formed during the sample dialysis process of lowering the concentration of NaCl from 300 to 150 mM) remained stable after being changed to a buffer with around 350–450 mM NaCl. This shows that these supramolecular assemblies are resistant to ionic strength conditions much higher than the physiological, suggesting that electrostatic forces are not essential for their cohesion.

**Figure 8:**
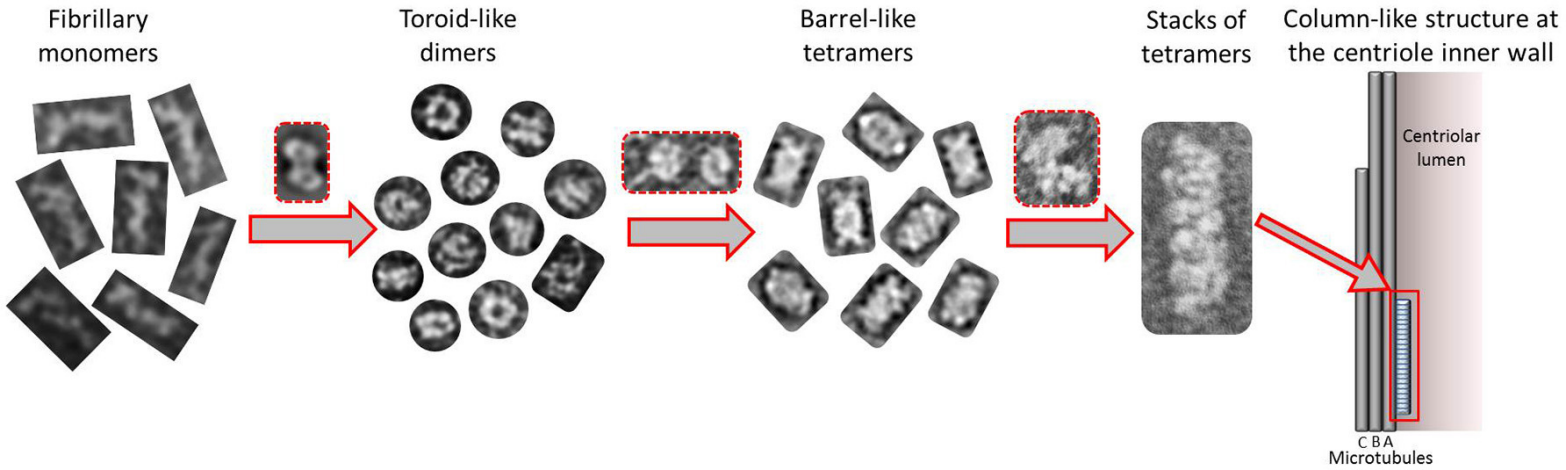
**Tentative model suggested for the progressive self-assemble of CPAP into gradually higher oligomeric subcomplexes until the formation of a modular, elongated structure**. The highly flexible CPAP monomers may dimerize by meshing into a globular toroid-like complex, which in turn could dimerize again resulting in the formation of a hollow, barrel-like tetrameric structure. Finally, stacks of tetramers may form linear arrangements of variable length with a periodicity close to 8 nm, which could also correspond to those structures known to exist at the inner walls of the centriole. Red dashed lines highlight putative intermediate states between one oligomeric state and the next one.

Taking together all the data presented in this work, we propose a putative model for the formation of higher order modular filaments of CPAP (Figure 8), where first, the highly flexible monomer strings of *Hs*CPAP^897−1338^ dimerize acquiring a globular and more stable toroidal structure. Then, dimers of *Hs*CPAP^897−1338^ would further dimerize into a tetrameric structure, which, in turn, could act as a somewhat flexible building block for larger, modular and elongated rope-like supramolecular structures with an axial periodicity around 8 nm. Finally, we propose that these modular rope-like structures formed *in vitro* could correspond to the column-like structures observed in the inner-walls of the centriole. Further studies are needed to establish the functional role of the different oligomers of *Hs*CPAP^897−1338^ (e.g., studying their interactions with reported protein partners of CPAP).

The suggested tentative organization of the G-box and CC4/CC5 domains on opposite sides of the putative tetramer structure of *Hs*CPAP^897−1338^ would confer to it a structural (and therefore functional) polarity, which, in turn, would also apply to the modular, higher order rope-like complexes. However, further work labeling the different domains of CPAP and the respective structural studies at quasi-atomic resolution must be carried out to unambiguously determine the domain organization of the monomers within the different complexes.

The presented work reinforces the idea that CPAP forms organized higher order structures allowing it to act as a scaffold that connects PCM proteins and complexes with the nascent centriole (Gopalakrishnan et al., 2011; Hatzopoulos et al., 2013; Zheng et al., 2014), contributing to the progression of procentriole assemble and elongation (Tang et al., 2009). The precise mechanism by which CPAP goes from one oligomeric state to a different one, as well as the exact biological role of each of these complexes, is a matter of future studies. Mutations within the region between residues 897 and 1338 of *Hs*CPAP, which impair its ability to associate with other proteins (Cottee et al., 2013; Gabriel et al., 2016), are associated with MCPH (Leal et al., 2003) and SCKS (Al-Dosari et al., 2010) diseases, thus the present study may provide novel structural clues that could help guide new works aimed to reach future biomedical applications. Naturally, these initial results must be complemented with structural data at quasi-atomic resolution (e.g., using cryo-EM methods) as well as functional studies. For example, a comparative study of the oligomeric behavior between wild-type *Hs*CPAP^897−1338^ and protein constructs containing point mutations or deletions associated with MCPH or SKCS syndromes (Bond et al., 2005; Al-Dosari et al., 2010; Gabriel et al., 2016), would provide valuable information to better understand some of the molecular basses of these clinical disorders.

Our results showing that *Hs*CPAP^897−1338^ displays a diverse and dynamic oligomeric behavior lay the foundation not only for future structural works, but also for new mechanistic/functional studies of this critical factor in centrosome formation and cilium disassembly and, consequently, in cell biogenesis.

## Accession numbers

The EM maps of the putative dimer and the putative tetramer of *Hs*CPAP^897−1338^ have been deposited in the Worldwide Protein Data Bank (wwPDB) (http://www.wwpdb.org/) with accession codes EMD-8283 and EMD-8288, respectively.

## Author contributions

AA and JC conceived the project and designed the work. AA carried out and designed most experiments and sample preparation. GBM assisted the protein purification experiments. AA, SD, and DG carried out the EM data collection. AA, CS, and JC performed the image analysis and 3D reconstruction of HsCPAP^897−1338^ complexes. AA and JC wrote the paper with significant inputs from GM, CS and TT. All authors reviewed the results, provided critical comments and input, proof- read, and approved the manuscript.

### Conflict of interest statement

The authors declare that the research was conducted in the absence of any commercial or financial relationships that could be construed as a potential conflict of interest.
