# Supplementary material for: Electron Microscopy Structural Insights into CPAP Oligomeric Behavior: A Plausible Assembly Process of a Supramolecular Scaffold of the Centrosome
Source: Front Mol Biosci. 2017 Mar 27;4:17. doi: 10.3389/fmolb.2017.00017 (PMC5366329; doi:10.3389/fmolb.2017.00017)
Supplement: Supplementary file 1 [file DataSheet1.pdf]

Supplementary Material

Electron microscopy structural insights into CPAP oligomeric behavior: A plausible assembly process of a supramolecular scaffold of the centrosome

Ana Lucia Alvarez-Cabrera[1, 2, *], Sandra Delgado Bermudez[3], David Gil Carton[3], Gulnahar B. Mortuza[4], Guillermo Montoya[4], Carlos Oscar S. Sorzano[1], Tang K. Tang[5], Jose Maria Carazo[1].

[1] Biocomputing Unit, Centro Nacional de Biotecnología – CSIC, Campus de Cantoblanco, Darwin 3, 28049 Madrid, Spain.

[2] Molecular and Computational Biology Program, Department of Biological Sciences, University of Southern California, Los Angeles, CA 90089, USA.

[3] Structural Biology Unit, CIC bioGUNE, Parque Tecnológico de Bizkaia Edificio 800, 48160 Derio, Spain.

[4] Protein Structure and Function Program, Novo Nordisk Foundation Center for Protein Research, Faculty of Health and Medical Sciences, University of Copenhagen, Blegdamsvej 3B, 2200 Copenhagen, Denmark.

[5] Institute of Biomedical Sciences, Academia Sinica, Taipei 11529, Taiwan.

*Correspondence: Dr. Ana Lucia Alvarez-Cabrera.
Affilation address: alalvarez@cnb.csic.es
Present address: analucia@usc.edu

Supplementary Table 1. Scores for the *Hs*CPAP domain models predicted by I-TASSER

*Hs*CPAP domain	Template PDB	C-score*	TM-score¥
CC4/CC5	2CH7	-1.18	0.806
CCGb-linker	4JIO	-2.63	0.603
G-box	4BXR	-1.05	0.794

*C-score: Confidence score for estimating the quality of predicted models by I-TASSER. Typically in the range of [-5,2], where a higher value signifies a model with a high confidence and vice versa.

¥ TM-score: Scale for measuring the structural similarity between two structures. A value >0.5 indicates a model of correct topology and a value <0.17 means a random similarity.

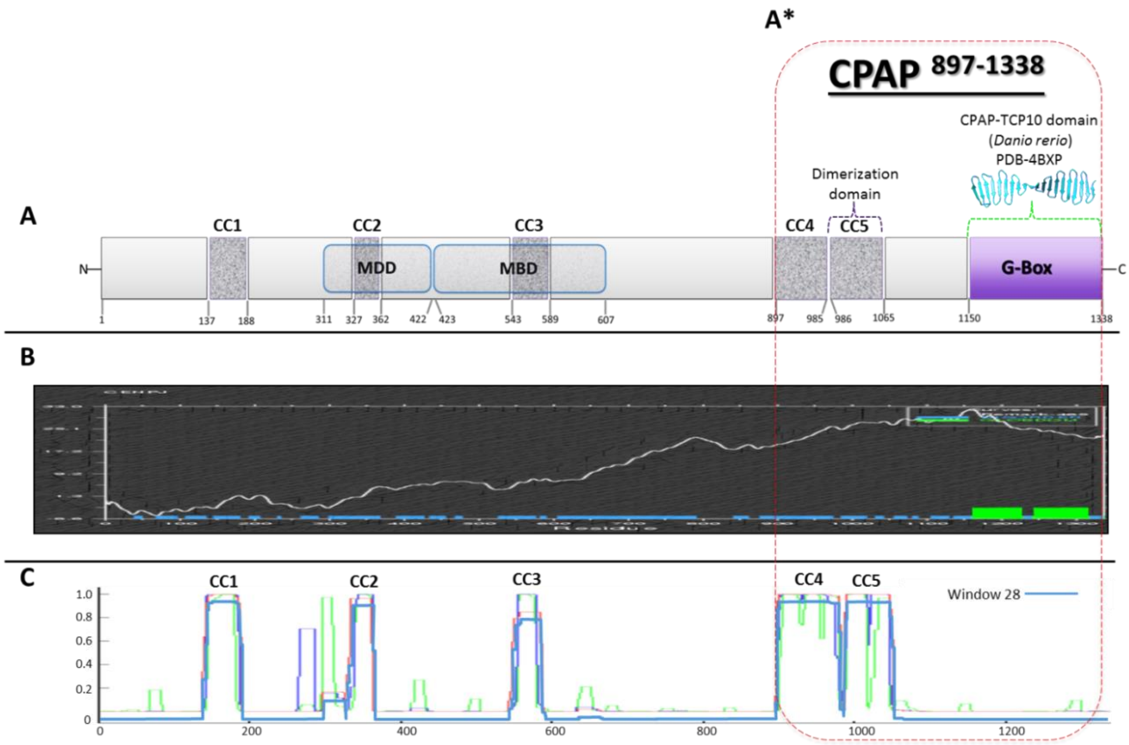

Supplementary Figure 1. Structural domains in human CPAP protein. (A) Schematic representation of the structural domains in *Hs*CPAP full-length protein. Dark gray boxes represent the five predicted CC (labelled as CC1, CC2, CC3, CC4 and CC5), being CC5 the oligomerization domain of CPAP; light gray corresponds to unstructured regions and the purple box refers to the G-box domain, whose crystallographic structure was already solved in *Danio rerio* (PDB-4BXP); MDD: tubuline-dimer binding domain and MBD: Microtubule binding domain. **(A*)** Red dashed lines demark the *Hs*CPAP$^{897-1338}$ construct. **(B)** In silico prediction of globular and disordered regions of proteins *Hs*CPAP (full-length), obtained using the program GlobProt2. The green boxes show the predicted globular domains, whereas the blue boxes correspond to disordered regions of the protein. **(C)** Probability prediction of five CC structures in CPAP, calculated with the software COILS.

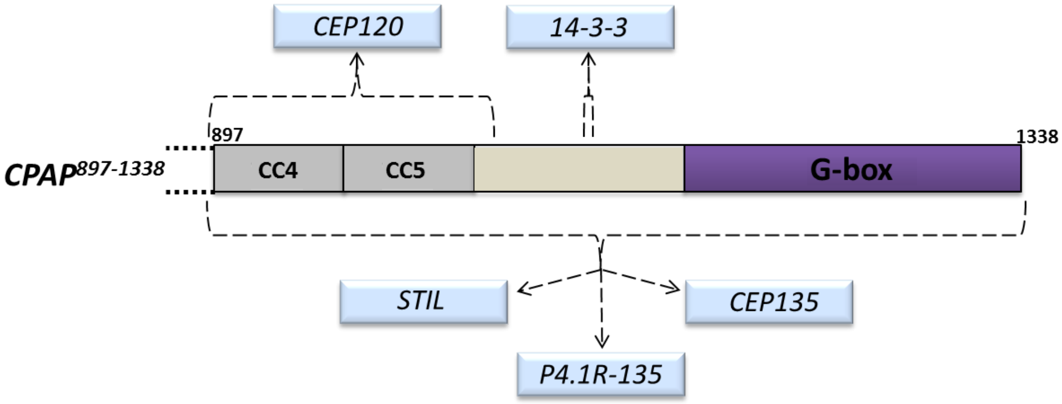

Supplementary Figure 2. *Hs*CPAP$^{897\text{-}1338}$ interacting partners. Approximate regions within *Hs*CPAP$^{897\text{-}1338}$ (demarked by the corresponding slashed curly brackets) that have been reported to interact with other centrosomal proteins (light-blue boxes).

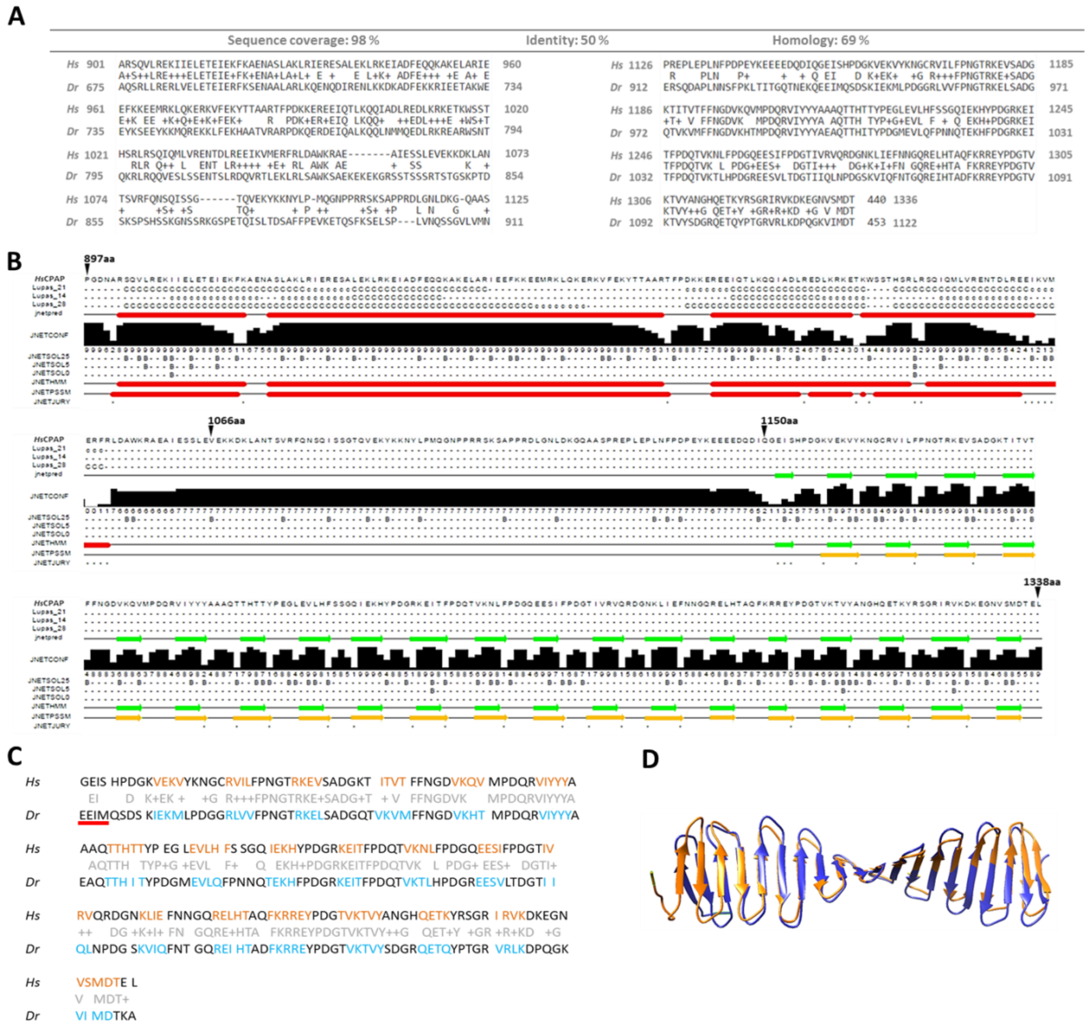

Supplementary Figure 3. Secondary structural prediction of HsCPAP897-1338. (A) Sequence alignment (protein BLAST) of HsCPAP897-1338 (Hs) and the equivalent domains in D. rerio CPAP (Dr). **(B)** The sequence includes the CC4/CC5 region (residues 897-1065), the CCGb-linker (residues 1066-1149) and the G-box (residues 1150-1338). The annotation bars below the alignment are as follows:
1) ***Lupas_21, Lupas_14, Lupas_28***: CC predictions for the sequence. These are binary predictions for each location. 2) ***JNETSOL25, JNETSOL5, JNETSOL0***: Solvent accessibility predictions - binary predictions of 25%, 5% or 0% solvent accessibility. 3) ***JNetPRED***: The consensus prediction. α-helices (*red tubes*) and β-sheets (*green arrows*). 4) ***JNetCONF***: The confidence estimate for the prediction. High values mean high confidence for prediction of α-helices (*red tubes*) and β-sheets (*green arrows*). 5) ***JNetALIGN***: Alignment based prediction. α-helices (*red tubes*) and β-sheets (*green arrows*). 6) ***JNetHMM***: HMM profile based prediction. α-helices (*red tubes*) and β-sheets (*green arrows*). 7) ***Jpred***: Jpred prediction. α-helices (*red tubes*) and β-sheets (*green arrows*). 8) ***JNETPSSM***: PSSM based prediction. α-helices (*red tubes*) and β-sheets (*orange arrows*). 9) ***JNETFREQ***: Amino Acid frequency based prediction. α-helices (*red tubes*) and β-sheets (*green arrows*). 10) ***JNETJURY***: A '*' in this annotation indicates that the JNETJURY was invoked to rationalise significantly different primary predictions. **(C)** Sequence alignment of the G-box domain from H. sapiens (Hs) and D. rerio (Dr), showing a comparison of the beta-sheet positions for both the I-TASSER atomic model prediction for Hs (labelled in orange) and the already

known structure from the PDB-4BXR for Dr (labelled in blue). The part corresponding to the Dr sequence underlined in red, is not resolved in the crystallographic structure. **(D)** Superimposed atomic structures of PDB-4BXR (*blue*) and Hs G-box I-TASSER model (*orange*).

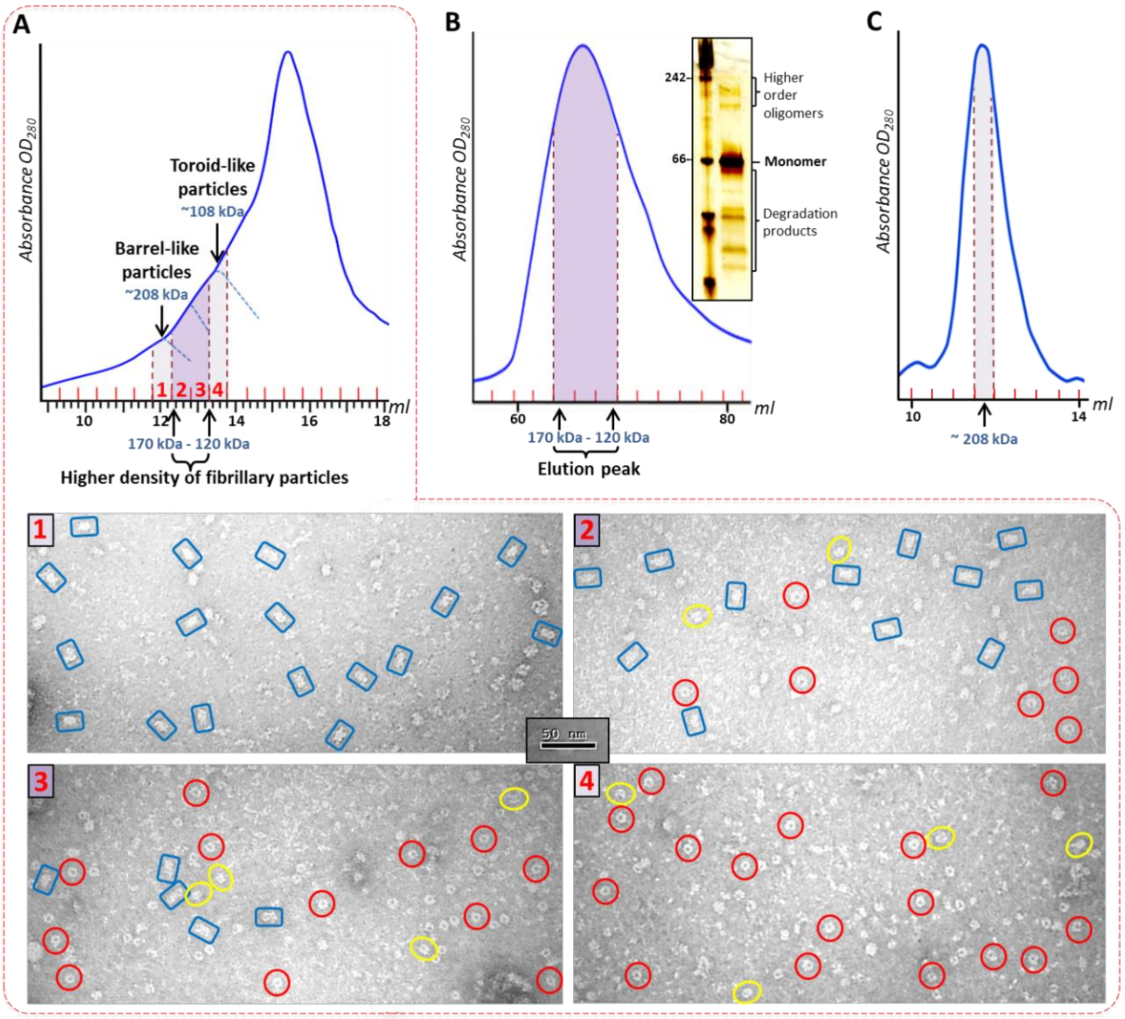

Supplementary Figure 4. Elution profile of size-exclusion chromatography (SEC) purification runs of *Hs*CPAP$^{897\text{-}1338}$ where different oligomeric forms were identified using NS-EM. (A) Chromatographic elution profile of a SEC column (Superdex 200 10/300) purification. Blue dashed lines mark three fused peaks under the graphic, where three main forms of the protein (fiber-, toroid- and barrel-like particles) were identified by NS-EM. Fractions 1 to 4 (*labeled with red numbers*) showed different levels of particle heterogeneity (*panels 1 to 4 images correspond with the number of each fraction*). Each of the three demarked areas under the curve contains the eluted fractions where the highest concentration of each of the three different types of particles was observed, as indicated by their respective label in the chromatogram and shown in the EM image of each of the analyzed fractions. Fraction 1 (*panel 1*) is clearly populated by barrel-like particles (highlighted by blue rectangles), while fraction 4 (*panel 4*) contains mostly what seems to be top and side views of toroid-like particles (highlighted by red circles and yellow ovals, respectively). Fractions 2 and 3 present a mixture in different proportions of the barrel- and toroid-like particles. The higher concentration of fiber-like particles present in fractions 2 and 3 (*panels 2 and 3, respectively*), make the background of their respective EM images to appear as a more dense mottled pattern compared with the background of fractions 1 and 4, which seem to contain lower amount of the flexible strings. SDS-PAGE analysis showed that

fractions eluted at the prominent absorbance peak around 15.5 ml, corresponded to degradation products of the protein (data not shown). **(B)** Elution profile (*left*) of a SEC column (Superdex 200 16/60) purification and Native–PAGE (*right*) of the elution peak (between 120-170 kDa), which was enriched by flexible fibrillary particles (observed by NS-EM). Native-PAGE (silver staining) shows that most of the protein is in a monomeric state (thick band close to the 66kDa marker), although there are some minority bands (over the 66kDa marker) corresponding to protein oligomers; some other lower bands show protein degradation products (under the 66kDa marker). CPAP identity of the bands was confirmed by MS analysis. **(C)** Elution profile of a SEC column (Superdex 200 10/300) purification, showing a peak at an apparent MW of ~208 kDa (compatible with a tetramer). NS-EM analysis of the peak fraction showed the same barrel-like particles observed to elute at ~208 kDa in the SEC run presented in panel **A**.

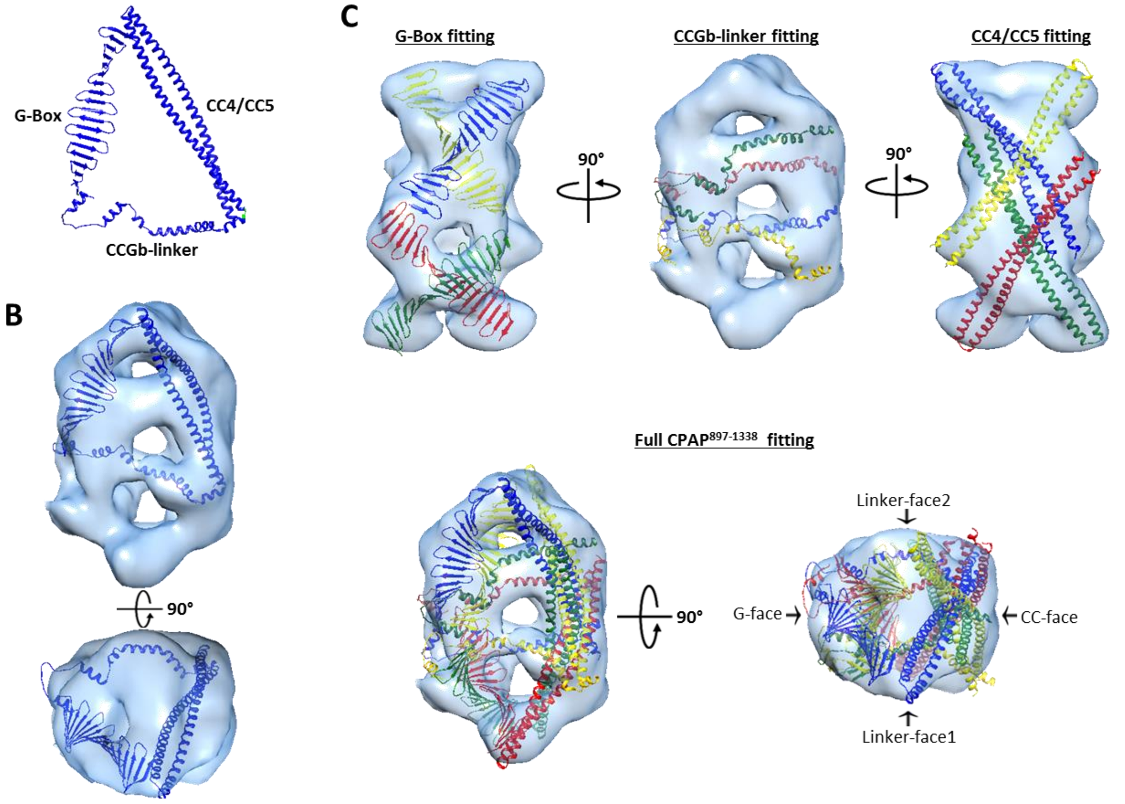

Supplementary Figure 5. Tentative fitting of four copies of a triangular conformation of the proposed atomic structure of *Hs*CPAP$^{897\text{-}1338}$ into the 3D map of the putative *Hs*CPAP$^{897\text{-}1338}$ tetramer. (A) Proposed triangular conformation of *Hs*CPAP$^{897\text{-}1338}$ monomer, using the obtained atomic models of the CC4/CC5 and CCGb-linker domains and the X-ray crystallographic structure of the G-box (PDB-4BXP). **(B)** Fitting of one copy of the complete monomer model into the 3D volume, where two different views of the EM density map of the complex are shown in translucent blue. **(C)** For clarity, in these figures the three structural domains of each of four fitted *Hs*CPAP$^{897\text{-}1338}$ monomers are painted with a different color. In the first row there are shown different views of the density map with the fitting of four copies of each of the structural domains, and in the second row it is shown the fitting of four copies of the complete monomer model into the 3D map. Abbreviations used for labeling different faces of the figure in the panel at the bottom right corner are: G, G-box; Linker, CCGb-linker and CC, CC4/CC5.
